# Future Leader to Watch – Fabrizio Alberti

**DOI:** 10.1242/bio.057679

**Published:** 2020-12-03

## Abstract

Future Leader to Watch is a series of interviews with the first authors of a selection of Reviews published in Biology Open, helping early-career researchers promote themselves alongside their papers. Fabrizio Alberti is first author on ‘Recent developments of tools for genome and metabolome studies in basidiomycete fungi and their application to natural product research’, published in BiO. Fabrizio is a Leverhulme Trust Early Career Fellow (Senior Research Fellow) at the School of Life Sciences and Department of Chemistry, University of Warwick, UK, investigating the discovery and biosynthetic characterisation of natural products made by fungi and bacteria.


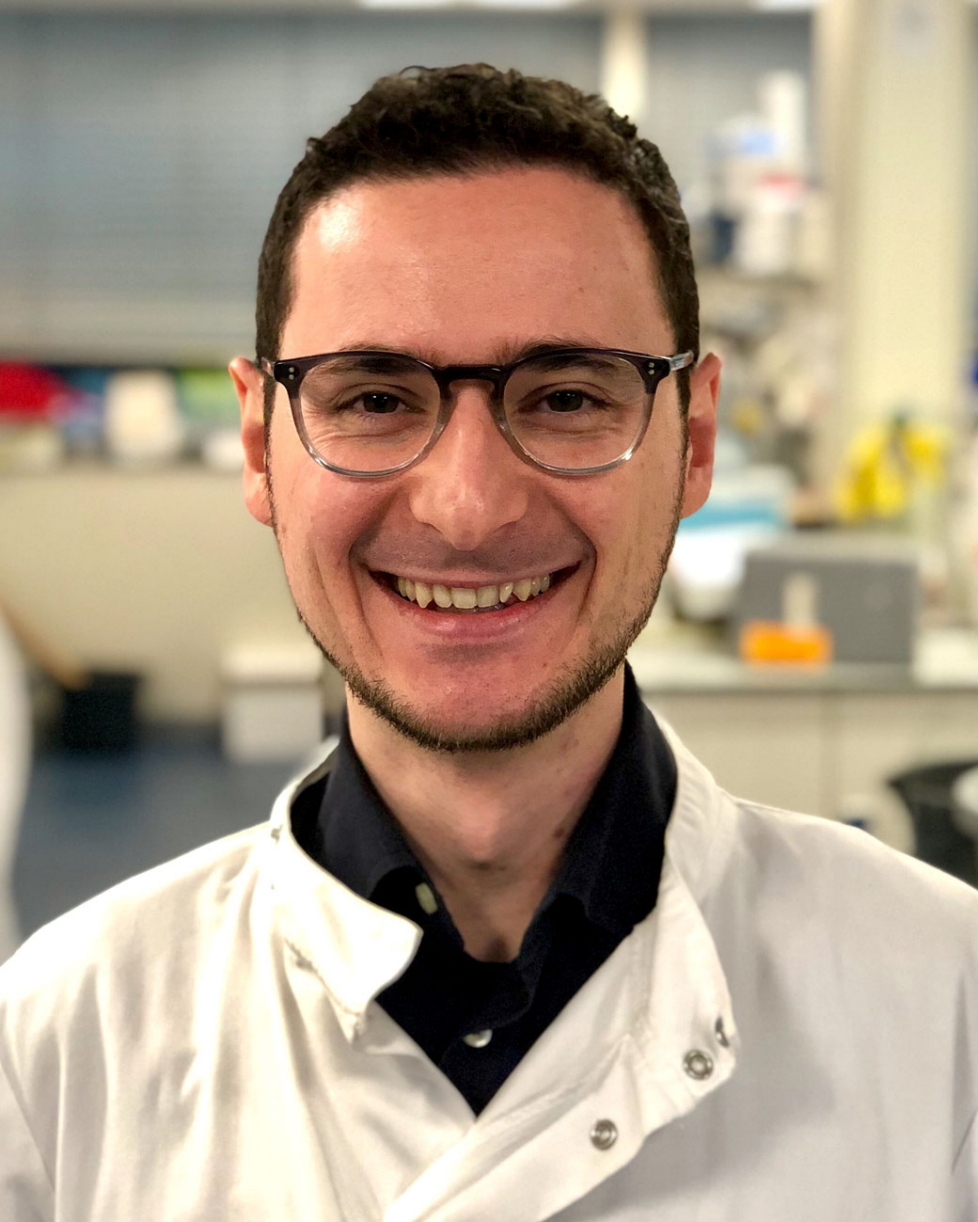


**Fabrizio Alberti**

**What is your scientific background and the story of how you got to where you are today?**

I have always been fascinated by microbiology and molecular biology, since my early days as an undergraduate student at the University of Perugia (Italy). During my PhD at the University of Bristol (UK) I studied the biosynthesis of an antibiotic molecule made by basidiomycete fungi, thus learning not only methods for the genetic engineering of microorganisms but also analytical chemistry techniques. In my postdoctoral work at the University of Warwick I decided to apply the skills that I had acquired in my PhD to a different topic: the discovery of natural products made by *Streptomyces* bacteria. I am currently working on the biosynthetic characterisation of bioactive metabolites made by fungi, with particular interest in terpenoid and meroterpenoid compounds.

**What is the most important take-home message of your Review?**

Basidiomycetes are fungi that can make bioactive natural products. However, they have large and complex genome structures and they are difficult to work with, as a consequence of their slow growth rate compared with other fungi and bacteria. Due to this, secondary metabolite study has lagged behind in basidiomycete fungi, with researchers more often focusing on their close relatives, ascomycetes. Recent developments in tools for genome and metabolome studies have taken place that can be applied to basidiomycetes, including on long-read whole-genome sequencing, CRISPR/Cas9-mediated engineering, tools for heterologous expression and molecular network analysis. These technologies are now allowing researchers to more easily study natural products made by basidiomycetes.

**What has surprised you the most while researching this Review?**

I was impressed by the efforts that researchers made in recent years to develop CRISPR/Cas9-based protocols for the engineering of basidiomycete fungi. As it is often the case in the fungal biology field, CRISPR/Cas9-based toolkits were first developed for use in ascomycetes, but now they are also being optimised for the more complex mushroom-forming basidiomycete species.

**What do you feel is the most important question that needs to be answered to move the field forward?**

I think that much still needs to be done to understand the complex architecture of basidiomycete genes. For example, the prediction of promoters and introns in this group of fungi is still inaccurate compared to other organisms. Efforts need to be done by researchers who work in this field to make transcriptomic and proteomic data publicly available and ultimately develop better bioinformatic predictions of basidiomycete genes.

**What changes do you think could improve the professional lives of early-career researchers?**

I think that we all appreciate that there is much more to science than simply working at the bench. From a professional point of view, I believe that early-career researchers would benefit immensely if science communication, manuscript and grant writing courses were an integral part of their graduate studies.

“I think that we all appreciate that there is much more to science than simply working at the bench.”

**What's next for you?**

My next research goal is to elucidate the biosynthetic pathway of selected fungal bioactive natural products using our recently developed yeast heterologous expression platform. My next career goal is to secure another personal fellowship and/or a lectureship position that will allow me to continue my research and to focus on longer term projects.
